# Sleep-Associated Torsades de Pointes: A Case Report

**DOI:** 10.5811/cpcem.2016.10.31352

**Published:** 2017-01-23

**Authors:** Guy Carmelli, Ian S. deSouza

**Affiliations:** SUNY Downstate University Hospital of Brooklyn, Kings County Hospital Center, Department of Emergency Medicine, Brooklyn, New York

## Abstract

Torsades de Pointes (TdP) is a polymorphic ventricular tachycardia that occurs in the presence of an acquired or congenital long QT syndrome (LQTS). We present the case of a 57 year-old man with end-stage renal disease on methadone maintenance in which there occurred multiple episodes of TdP during sleep. The patient was found to have a QTc interval of 548 milliseconds, and the dysrhythmia was successfully treated with isoproterenol infusion and methadone substitution. It is surmised that the patient had a multifactorial, acquired LQTS that during somnolence, reached a critical threshold of QT prolongation to lead to the development of TdP.

## INTRODUCTION

Sudden cardiac death (SCD) is a major cause of death worldwide, with a reported incidence in the U.S. of more than 400,000 cases per year. One uncommon cause of SCD is torsades de pointes (TdP), defined as polymorphic ventricular tachycardia occurring in a patient with an acquired or congenital long QT syndrome (LQTS).^1^ TdP accounts for fewer than 5% of SCD cases, and death presumably occurs from its degeneration into ventricular fibrillation.^1^ Medications with QT-prolonging effects are the most frequent cause of acquired LQTS, which is estimated to occur with approximately 2–3% of all prescriptions written.^2^,^3^ Another cause of acquired LQTS involves electrolyte disturbances, including hypokalemia, hypocalcemia, and hypomagnesemia.^4^,^5^ Furthermore, LQTS may be a result of cardiac abnormalities such as bradycardia with atrioventricular (AV) block, congestive heart failure, ischemic heart disease, rheumatic heart disease, myocarditis, and mitral valve prolapse.^4^,^5^ This case report discusses a patient in a normal physiologic state of sleep that may have contributed to the development of TdP associated with an acquired LQTS.

## CASE REPORT

A 57 year-old man with past medical history of hypertension, diabetes mellitus, dyslipidemia, and end-stage renal disease was transferred from his dialysis center after suffering a cardiac arrest. According to the center’s staff, he experienced ventricular fibrillation for which he was immediately defibrillated with an automated external defibrillator. During the chest compressions that followed, the patient promptly regained consciousness. After the successful resuscitation, he was transferred to the emergency department (ED) by emergency medical services.

Upon arrival to the ED, the patient had no complaints. He recounted that during his dialysis session, he “was watching TV, got tired and fell asleep, and was woken up by the shock.” He reported no prodromal symptoms prior to the event. His maintenance medications included simvastatin, clonidine, insulin, and methadone. The patient had an elevated blood pressure of 160/84, heart rate of 85, respiratory rate of 20, and temperature of 97.6 °F. His oxygen saturation was 97% on nasal cannula at three liters of oxygen per minute. Mental status was normal, and skin was normally perfused. Cardiorespiratory examination was normal except for the presence of a dialysis catheter in the right side of the chest. Pupils were 1–2 millimeters bilaterally.

**Image 1:**
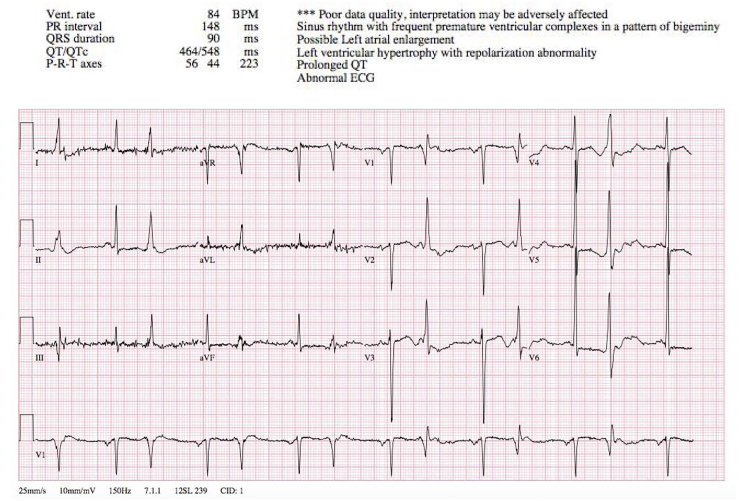
Initial ECG in the emergency department showing prolonged QTc of 548 msec. *ECG,* electrocardiogram

Aspirin 325 mg was given orally. The initial electrocardiogram (ECG) revealed sinus rhythm with junctional bigeminy, an occasional premature ventricular complex, left ventricular enlargement, and a significantly prolonged QTc of 548 milliseconds (Image 1). Continuous cardiac telemetry was initiated, and electrophysiology consultation was sought with the plan of hospital admission to the coronary care unit (CCU). Serum potassium was measured at 3.6 mEq/L and magnesium was 2.0 mg/dL, both of which were within the normal ranges.

Approximately three hours into the ED course, the patient lost consciousness, and the monitor demonstrated ventricular fibrillation (VF). Immediate defibrillation at 200J was performed, and the patient quickly regained consciousness during subsequent chest compressions. A repeat examination was unchanged. Due to presumed recurrent VF, metoprolol 5 mg was given intravenously to treat possible electrical storm. Minutes later, the physician at the bedside observed that the patient had become somnolent. The monitor showed sinus bradycardia, which was succeeded by polymorphic ventricular tachycardia. The physician again successfully defibrillated the patient. The diagnosis of TdP was now presumed, and a magnesium infusion of 1 gram was administered over 30 minutes. The patient experienced one more identical arrest sequence, and upon recommendation by cardiology, a lidocaine infusion was started. The patient was transferred to the CCU.

**Image 2:**
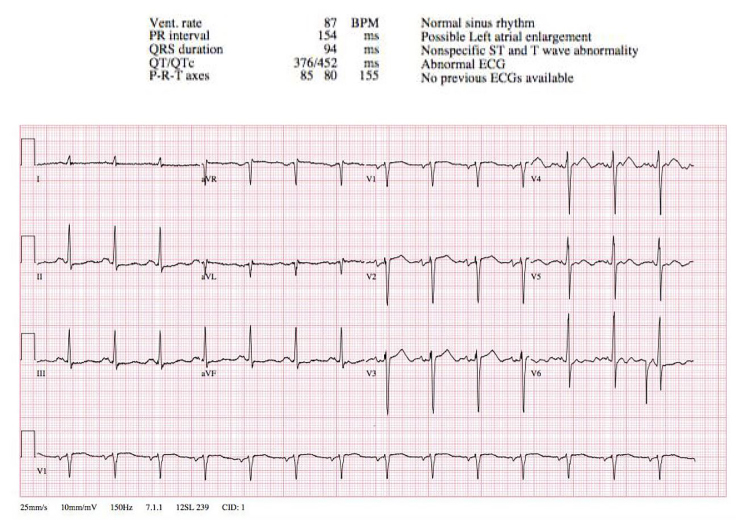
Repeat ECG in the coronary care unit after methadone cessation showing normal QTc of 452 msec. *ECG,* electrocardiogram

During the CCU course, methadone dose was slowly reduced. In spite of the lidocaine infusion, the patient continued to have occasional bradycardia episodes during somnolence. As a result, the lidocaine infusion was discontinued, and an isoproterenol infusion was initiated. The patient’s QTc remained prolonged on the lower methadone dose, so the drug was discontinued and substituted with buprenorphine/naloxone. The patient had no more ventricular fibrillation episodes, and a repeat ECG revealed a QTc of 450 milliseconds (Image 2). Serial troponins and electrolytes continued to be normal, and an echocardiogram indicated systolic dysfunction with an ejection fraction of 30–35%. Additional hospital records included the results of a cardiac catheterization three weeks prior that showed non-obstructed coronary arteries. The isoproterenol infusion was gradually tapered, and the patient was fitted for a wearable defibrillator. He was discharged on hospital day 12 with continued outpatient treatment for opioid dependence and plan for possible automated implantable cardioverter-defibrillator placement.

## DISCUSSION

### Pathophysiology

The QT interval represents a portion of the cardiac action potential, mainly ventricular repolarization.^2^,^6^ If a factor such as a medication or electrolyte abnormality was to delay repolarization, this would manifest as a prolonged QT interval on the ECG. During such prolongation of the repolarization phase, there is a destabilization in membrane channels that may give rise to an after depolarization.^7^,^8^ An after depolarization is an oscillation of the membrane potential of one cell that occurs as a result of the upstroke of an action potential of a nearby, stimulated cell.^7^,^8^ If an afterdepolarization is strong enough to bring the cell membrane to its threshold potential, it will result in a depolarization. This depolarization, when it occurs during repolarization, is commonly known as the “R on T phenomenon,” the inciting event for the TdP dysrhythmia.^8^ Furthermore, since this rhythm is a response to a preceding impulse, it is referred to as a triggered activity.^8^

### Back to Our Case

This patient had numerous risk factors for TdP. He presented with a significantly prolonged QTc and then experienced episodes of sinus bradycardia. The patient had both a prolonged QT and QTc, suggesting that other factors, beyond the heart rate, were contributors to delayed repolarization. Methadone is one of many medications that have been shown to directly influence cardiac repolarization,^6^,^9^,^10^ and even low doses of methadone (<100mg) have been shown to cause QT prolongation.^6^,^9^,^10^

There is an inverse relationship between heart rate and duration of repolarization.^2^ Slow heart rates, even benign, sinus bradycardia, are characterized by longer repolarization times and therefore longer QT intervals.^2^ QT prolongation may also result from drug-induced bradycardia or pathological causes such as high-grade AV blocks or sick sinus syndrome.^2^,^11^ In this case, TdP was observed when the patient fell asleep, and somnolence itself is characterized by bradycardia.^12^,^13^,^14^ Polysomnography of healthy individuals has detected bradycardia to as low as 30 beats per minute and sinus pauses averaging from 2–11 seconds, both of which occur during early non-REM and REM sleep stages.^12^,^13^,^14^ This is hypothesized to be a result of either decreased adrenergic tone or increased vagal stimulation that occurs during physiologic sleep.^12^,^13^ Holter electrocardiography of healthy subjects has also shown that *both* the QT *and* the QTc intervals increase during somnolence, along with episodes of bradycardia and sinus pauses during sleep.^15^ It is therefore suggested that heart rate changes may not be the only contributor to the QT prolongation during sleep, and that sleep itself may be an independent, QT-prolonging factor.^15^ While we were not able to obtain a rhythm strip during each episode that captured the exact onset of TdP, one of the authors directly observed the dysrhythmic event to be remarkably coincident with somnolence. It is therefore reasonable to suspect that the recurrent dysrhythmia occurred due to a further prolonged QT as a result of sleep, directly and/or through sleep-associated bradycardia or sinus pause.

Lastly, the procedure of hemodialysis, interestingly, can lead to QT changes, aside from the electrolyte abnormalities alone.^16^ Prolongation of the QT and decreased adaptability of QT interval to variations in heart rate have been reported to occur during dialysis sessions.^16^ Sudden cardiac death may occur, and if so, occurs most often in the immediate period after a hemodialysis session during which altered ventricular repolarization can persist for several hours.^16^ Although our patient’s electrolytes were found to be within normal limits at time of presentation, the lingering cardiac effects of his recent hemodialysis session may have played a role in the development of TdP.

## CONCLUSION

This case describes an interesting presentation of TdP, presumably due to a variety of factors that resulted in an acquired long QT syndrome. There are numerous risk factors for the acquired form of LQTS, with novel causes being reported each year.^17^,^18^,^19^ However, what distinguished this case were the recurrences of TdP during somnolence. While previous studies have suggested a theoretical mechanism for TdP during sleep, we are not aware of any cases in the literature.^1^,^7^,^13^ This patient ultimately did well with judicious treatment including prompt electrical therapy, magnesium, isoproterenol, and methadone cessation.
